# Oxidative Stress as Cause, Consequence, or Biomarker of Altered Female Reproduction and Development in the Space Environment

**DOI:** 10.3390/ijms19123729

**Published:** 2018-11-23

**Authors:** Jon G. Steller, Jeffrey R. Alberts, April E. Ronca

**Affiliations:** 1 Division of Maternal Fetal Medicine, Department of Obstetrics & Gynecology, University of Colorado School of Medicine, Aurora, CO 80249, USA; 2 Department of Psychological & Brain Sciences, Indiana University, Bloomington, IN 47405, USA; alberts@indiana.edu; 3 Space BioSciences Division, NASA Ames Research Center, Moffett Field, CA 94305, USA; april.e.ronca-1@nasa.gov; 4 Department of Obstetrics & Gynecology, Wake Forest School of Medicine, Winston-Salem, NC 27101, USA

**Keywords:** oxidative stress, cosmic radiation, microgravity, hind-limb unloading, spaceflight, reproduction, female, pregnancy

## Abstract

Oxidative stress has been implicated in the pathophysiology of numerous terrestrial disease processes and associated with morbidity following spaceflight. Furthermore, oxidative stress has long been considered a causative agent in adverse reproductive outcomes. The purpose of this review is to summarize the pathogenesis of oxidative stress caused by cosmic radiation and microgravity, review the relationship between oxidative stress and reproductive outcomes in females, and explore what role spaceflight-induced oxidative damage may have on female reproductive and developmental outcomes.

## 1. Introduction

Orbital spaceflight is now a continuous reality, and deep space exploration, along with extra-Earth colonization, have become the aspirational activities of NASA and space agencies in countries besides the U.S., as well as numerous private entities. Determining the long-term physiological responses and adaptation to the space environment and re-adaptation to the environmental conditions on Earth is critically important to the safety and health of the next coterie of astronauts. Further, understanding the impact of extraterrestrial environments on human reproduction is vital to the success of space travel and colonization, as well for individuals returning to Earth. Indeed, this information will ultimately be critical for the survival of the species.

Remarkably little is known about the effects of microgravity, cosmic radiation, or other elements of spaceflight on mammalian reproductive physiology or developmental processes. One emerging area of particular interest to reproductive developmental investigators is oxidative stress and its potential effects on reproductive physiology and fitness in the context of long-duration spaceflight conditions. Significantly, both microgravity and cosmic radiation may be implicated in the tissue dysfunction caused by oxygen-free radicals and impaired reduction-oxygenation (redox) signaling [1]. Moreover, several lines of evidence implicate oxidative stress in the pathophysiology of numerous reproductive complications [2,3,4].

The present review begins with an initial examination of evidence for pathological aspects of reproductive physiology and oxidative stress associated with terrestrial mechanisms. There are a number of reproductive health concerns for both men and women in space which are potentially related to microgravity and/or stress (including sleep disruption). However, our focus here will be on the female body, because the maternal physiology of reproduction in space is an area of both long-standing interest and neglect. Our focus includes some basic aspects of offspring development. Reproductive success is, of course, the ultimate metric of reproductive physiology—the mammalian system, which is our main area of interest, comprises a series of perinatal phases during which offspring development is directly linked to maternal physiology and behavior. Understanding mammalian reproduction in relation to spaceflight requires that we go beyond the studies of the male reproductive organ system which is reviewed elsewhere [5,6,7]. In contrast to men, a small percentage (11%) of astronauts have been women; thus, reproductive changes during or post-flight have not been systematically studied in female astronauts [5]. This is our emphasis. We also consider how the extraterrestrial environment may lead to oxidative stress in non-reproductive tissues, and lastly, conjecture what role the space environment may play in oxidative damage that could impact fertility and pregnancy. Many of the effects, as well as some of their identified underlying mechanisms, may be considered as candidate biomarkers for reproductive perturbations. While no formal studies have been performed evaluating the latter association, this review also functions as a foundation for hypothesis generation with examples of future necessary endpoints to be explored at its conclusion.

## 2. Introduction to Oxidative Stress

Oxygen is critical for human life and many of the living systems on Earth. Nevertheless, oxygen may also be harmful to the same biological systems. The reactivity of oxygen promotes its participation in high-energy electron transfers critical for biologic redox reactions, such as those required during the synthesis of adenosine-5-triphosphate (ATP), as well in intracellular signaling pathways where it serves as a secondary messenger [8]. However, this reactivity also renders many biological molecules, like DNA, proteins, and lipids, vulnerable to oxidative damage from reactive oxygen species (ROS) when a free radical from the redox reaction does not reattach to the redox molecule or get neutralized by an antioxidant.

The term “oxidative stress” implies that the physiological balance between the creation of ROS and the ability to detoxify these molecules has been upset, leading to resultant stress and damage to cellular systems. Importantly, this can either indicate that there may be an abnormal elevation in ROS generation, or that there may be deficiencies in antioxidant defense systems. While ROS can serve as second messengers, be purposefully weaponized by our immune system to fight pathogens [9], or even prove beneficial to organisms through hormesis [10], oxidative damage is largely implicated in a legion of terrestrial pathologic processes, including neurodegenerative disease [11], heart disease [12], cancer [13], and disorders of pregnancy [14].

How this pathogenesis occurs depends on the type of system being affected. Because reactions to ROS are often diffusion-limited, their effect on cell function may depend upon the concentration of ROS and antioxidants present, as well as the type of cell they may be affecting. Well-established pathologic mechanisms include their ability to cause DNA strand brakes, DNA base damage, as well as disruption of cellular signaling pathways [1]. Furthermore, ROS can lead to failure of membrane fluidity and function, as well as activation of apoptosis, following lipid peroxidation of the plasma membrane or organelles [14]. However, what complicates our understanding of oxidative pathophysiology is that oxidative stress rarely occurs in isolation. Any insult leading to oxidative stress may also be causing other forms of cell stress—namely, endoplasmic reticulum (ER) stress—and may be disrupting other related signaling pathways.

### 2.1. Reactive Oxygen Species

ROS are termed “reactive” because they are oxygen species that contain at least one unpaired electron. However, the definition generally includes both free radicals themselves and their intermediates. Under physiological conditions, the most common oxygen-free radical is superoxide (O_2_^−^), which is predominantly produced in the mitochondria. During the transfer of electrons along respiratory chain enzymes, leakage of electrons onto molecular oxygen results in superoxide. The rate of formation can be increased both under conditions of hyperoxia, and hypoxia when the reduced availability of oxygen to act as the final electron acceptor in respiratory complex IV leads to improper electron accumulation [14]. Another source of superoxide is the ER, where electrons leak during the process of protein folding. This may be exacerbated by conditions of ER stress when continued attempts to refold misfolded proteins may lead to superoxide formation [15]. Other sources include xanthine dehydrogenase, which degrades purines, xanthine, and hypoxanthine to uric acid and leads to superoxide radical formation under hypoxic conditions, and nicotine adenine dinucleotide phosphate (NADPH) oxidase which is found in neutrophils and vascular cells, and activated by bacterial products and cytokines [16]. Uniquely, NADPH oxidase is also activated during pregnancy, and results in increased superoxide, especially in early pregnancy [17]. Finally, superoxide can also lead to interactions with nitric oxide to form peroxynitrite, which can lead to protein and DNA damage [18]. Other factors, including activation of redox-sensitive transcription, activation of protein kinases, opening of ion channels, lipid peroxidation, protein modifications, DNA damage, and a relationship with ER stress, are reviewed elsewhere [14].

### 2.2. Antioxidants

Antioxidants can be enzymatic or non-enzymatic, and are named as such due to how they inhibit oxidant attack on proteins, lipids, carbohydrates, and DNA by neutralizing free radicals. The most common enzymatic antioxidant is superoxide dismutase (SOD), which converts superoxide to hydrogen peroxide (H_2_O_2_) in both the mitochondria and cytosol of cells. Impaired action of this enzyme or its associated enzymes have been linked to oxidative stress [19]. Hydrogen peroxide can then be converted to water by other enzymes like catalase, peroxiredoxins, and/or glutathione peroxidases. These enzymes are extremely important for maintaining the delicate balance between superoxide and H_2_O_2_, because an imbalance favoring H_2_O_2_ may lead to reactions with endogenous transition metals via Fenton mechanics, resulting in formation of hydroxyl (OH*) ions. Though not mentioned in the ROS section above, these hydroxyl ions are some of the most damaging free radicals to cellular tissues [20,21]. Furthermore, animal studies involving over-expression of enzymatic antioxidants, such as catalase, have revealed a protective effect from neurologic sequelae, stemming from proton-induced irradiation [22,23,24].

Non-enzymatic antioxidants include ascorbate (vitamin C), tocopherols like α-tocopherol (vitamin E), and carotenoids like β-carotene, which all scavenge and neutralize free radicals [25,26]. Notably, antioxidant enzymes depend upon metal co-factors, which are capable of taking on different valences as they transfer electrons during redox reactions; thus, such metals as copper, zinc, manganese, iron, and selenium may also be considered to fall under the umbrella of “antioxidants” [27]. Further, polyphenols which can be found in a number of fruits and vegetables also serve as natural antioxidants [28].

## 3. General Considerations of Spaceflight and Oxidative Stress

Interplanetary spaceflight invites two major extraterrestrial exposures—cosmic radiation and microgravity. When exposure to spaceflight leads to pathophysiological outcomes, it is difficult or impossible to specify causal factors, for there are numerous candidates, most of which can act simultaneously or even jointly. Thus, general associations between oxidative stress and spaceflight are first discussed before evaluating evidence from ground-based analogs.

Spaceflight has been linked with oxidative damage in numerous non-reproductive tissues [1,29,30,31,32,33,34,35,36]. Because many of the findings were derived from post-flight evaluations, the stresses of reentry and terrestrial re-adaptation must be recognized as potential concomitant causes of oxidative stress. In fact, there are reports of increased excretion of isoprostane (a marker of oxidative stress) in human urine post-flight, after documenting stable levels of excreted isoprostane prior to their return [30,37]. Thus, it is hypothesized that there may be a post-flight increase in anabolic activity following spaceflight conditions, accompanied by reduced activity and caloric intake, in addition to downregulated antioxidant defense systems [30]. Lending further support for these hypotheses are two studies in which hair follicle samples collected post-flight from ISS crew members revealed decreased expression of numerous antioxidant genes [32], as well as decreased circulating serum antioxidants [31]. Other findings have included increased lipid peroxidation in human erythrocyte membranes [38], abnormal transcription of genes involved in ROS metabolism, antioxidative systems, and cellular response to oxidative stress [39], and a multitude of effects on human bone [35] and cardiovascular systems [40]. A recent, non-reproductive, organ-based approach to the evaluation of oxidative stress and its effects on bone loss, cardiovascular function, immune insufficiency and metabolism, and neurological impairment can also be found in this journal [36].

Given the limitations of human samples, rigorous analyses of oxidative stress have been conducted with rodents subjected to spaceflight. Findings have included increased cardiac gene expression of mitochondrial redox-related enzymes [41], increased hepatic gene expression of both ROS-scavenging and ROS-producing genes [42], increased ocular gene expression for enzymes involved in the regulation of ROS and the mitochondrial-apoptotic pathway [34], decreased enzymatic activity of numerous hepatic antioxidant enzymes [29,43], and increased production of lipid peroxidation products [44].

## 4. Microgravity and Oxidative Stress

### 4.1. General Pathophysiology of Microgravity

It is difficult or impossible to assess the specific effects of microgravity in isolation of other spaceflight factors. Similarly, when humans are the subjects, there are almost always deliberate countermeasures regularly provided during flight exposures. Nevertheless, it is now recognized that various biological tissues and systems react and adapt to microgravity differently. Initially, it is typical to observe prominent shifts in thoracic body fluids and a syndrome of space motion sickness. Additional anatomical and physiologic alterations include: anthropometric changes, significant cardiovascular changes that occur when venous pressures normalize secondary to elimination of hydrostatic gradients [45], small changes in lung capacities and tidal volume [46], loss of bone mineral density from weight-bearing bones [47], skeletal muscle atrophy [48], increased insulin resistance [49,50], modest sensorimotor changes [51,52,53], increased glomerular filtration rate and decreased sweating/insensible losses [54], and secondary effects, such as reduction in red blood-cell mass [55,56].

### 4.2. Oxidative Damage Associated with Ground-Based Microgravity Analogs

In non-human animals (usually rodents), the most established and widely-used method for simulating microgravity on Earth involves hindlimb unloading (HU) [57,58,59]. Typically, this model involves suspending rodents by the tail base to produce a 30-degree, head-down tilt position which prevents the hindlimbs from bearing weight. HU with head-down tilt produces the cephalad-directed fluid shifts observed in microgravity and the relative inactivity and immobilization that may contribute to decreased bone mineral density (BMD) and muscle loss [58]. HU has been used extensively to investigate musculoskeletal responses as well as cardiovascular adaptation, and a number of studies have evaluated oxidative stress in various tissues in this setting. In one study, HU was associated with elevated markers of oxidative stress in seven different tissues, including the brain, lung, pancreas, kidneys, intestine, heart, and liver, when compared with controls [60]. Other studies have revealed increased gene expression of ROS-scavenger enzymes in rat bone marrow [61], increased ROS in rat cerebral and carotid arteries [62], increased ROS in rat brain [63], and an increase in ROS, alterations in antioxidant defenses (increase in SOD, decrease in catalase, and glutathione peroxidase activities), decrease in non-enzymatic antioxidant scavenging capacity, and decrease in heat-shock proteins in rat muscle [64,65,66]. This has led investigators to hypothesize that a redox imbalance, leading to the activation of proteolysis and a massive oxidation of proteins, exacerbates muscle atrophy [67]; however, a causal role has not been established, nor have antioxidants been shown to reduce atrophy [68,69,70,71].

## 5. Cosmic Radiation and Oxidative Stress

### 5.1. General Pathophysiology of Cosmic Radiation

Sources of terrestrial radiation, such as x-rays, computed tomography (CT), nuclear energy, and atomic bombs result in exposure to x-ray and gamma irradiation—neither of which are encountered in space at levels of clinical significance. Clinically relevant forms of cosmic radiation include protons, heavy ions (i.e., Fe, Si, O), and the secondary neutron irradiation that occurs as protons bombard spacecrafts, habitats, or even impact the human body directly. Protons, heavy ions, and neutrons are distinct from x-ray and gamma irradiation in that they are all made up of particles, rather than “rays”, and transmit energy to cells differently. This is known as high-LET (linear energy transfer), in that these high-energy particles transfer energy along their entire path, leading to densely clustered hits which cause damage to proteins, lipids, cell membranes, extracellular matrix, DNA, and lead to oxidative stress [72,73,74].

### 5.2. Oxidative Damage Associated with Ground-Based Cosmic Radiation Analogs

As with ground-based microgravity analogs, numerous non-reproductive tissues have previously shown evidence of oxidative stress in the setting of simulated cosmic-radiation. Rodents exposed to high-dose proton or heavy ion irradiation have shown increased oxidative damage in the heart [75] and brain, leading to cognitive dysfunction [23,72]. Retinal endothelial cells evaluated after murine exposure to whole-body gamma-ray, proton, and oxygen ion radiation revealed elevated eNOS expression [76]. Further, increased activity of numerous genes involved with oxidative stress-related apoptotic pathways (Sod2, Sod 3, Gpx, Gpx2, Ucp3, Fmo2, and Noxa1) have also been found in rat retina following exposure to proton irradiation [77,78].

Less physiological studies using other sources of radiation, such as high-dose gamma irradiation, have also revealed increased ROS generation and lipid peroxidation in bone marrow [79,80,81]. Furthermore, increased expression of the oxidative defense gene, *NRF2*, which regulates cellular redox balance and carries a protective antioxidant response [82,83,84] has been altered in response to irradiation. In conditions of excess ROS, NRF2 promotes transcription of hundreds of antioxidant genes, including SOD1 [85]. Importantly, in addition to a number of other pathologies, NRF2 knock-out mice are predisposed to significantly increased bone loss in the setting of radiation exposure [86]. Lastly, both irradiation alone, and irradiation plus HU have been associated with diminished SOD1 and higher xanthine oxidase (XO; a pro-oxidant) protein content [1].

## 6. Oxidative Stress and Female Reproductive Physiology

The menstrual cycle is directed by a complex interplay of positive and negative feedback loops between the hypothalmus, anterior pituitary, and the ovaries, known as the “HPO” axis. These hormonal interactions govern two concomitant anatomical cycles occurring in the ovaries and uterus, which each have three phases. For the ovaries, these include: the follicular phase, ovulatory phase, and luteal phase; and for the uterus—the menstrual phase, proliferative phase, and secretory phase. For ovulation to occur, follicle-stimulating hormones (FSH) from the anterior pituitary drives ovarian follicle maturation. As the follicles (with eggs inside) grow, they create estradiol (E2). This increase in E2 drives endometrial growth and begins suppressing FSH. The follicle which is most sensitive to FSH will grow the best and win the maturation race, while the other competing follicles will then die off. Having an elevated E2 for greater than 50 hours causes the luteinizing hormone (LH) surge, which triggers ovulation and expulsion of the egg from the follicle. The follicle which secreted the egg then turns into the corpus luteum, and secretes progesterone (if the egg is fertilized) to help maintain an early pregnancy. If no pregnancy results, E2 and progesterone levels fall, the endometrium sheds, and FSH (no longer suppressed) will again trigger initiation of a new follicle maturation race [87].

ROS have been found to have physiological roles in almost every step in this pathway, including ovarian steroid biosynthesis, oocyte maturation, ovulation, formation of blastocysts, fertilization, implantation, luteolysis, and luteal maintenance [88,89,90,91,92]. Conversely, an imbalance in pro-oxidants and antioxidants can lead to a pathological role in each of these steps, and has been associated with endometriosis, polycystic ovarian syndrome, and infertility. Further, pregnancy makes this balance even more precarious by innately shifting it slightly towards a state of oxidative stress [93], which is evident in both the first trimester for placentation [3], and in the third trimester when there is increased activation of peripheral granulocytes, monocytes, and lymphocytes, all of which produce ROS [94]. However, any further pro-oxidative shifting can lead to a multitude of adverse pregnancy outcomes, including: miscarriage, recurrent pregnancy loss, embryopathy, pre-eclampsia, fetal growth restriction (FGR), preterm premature rupture of membranes (PPROM), and gestational diabetes (GDM) [4].

## 7. Oxidative Stress and Pregnancy

### 7.1. Oxidative Stress and Placentation

Humans have hemochorial placentas, denoting that the chorion is in direct contact with maternal blood, and trophoblast cells play an important role in this type of placenta. During placentation, trophoblast cells invade the myometrium and replace endothelial and smooth muscle cells in uterine spiral arterioles, which is believed to be critical for mediating the major increase in uteroplacental blood-flow to a normal pregnancy [95]. Interestingly, oxidative stress plays a physiological role in allowing for proper placentation to occur. It is believed that early placental development occurs in a relatively low-oxygen environment to protect the early embryo from ROS [3,96,97], and that once utero-placental circulation has been established, a three-fold rise in oxygen concentration will occur within the placenta [3]. This leads to elevations in ROS concentration in the syncytiotrophoblast, which has low concentrations of enzymatic antioxidants, and appears to preferentially trigger an apoptotic cascade in peripheral villi and help the placenta regress into a definitive discoid placenta [98,99,100]. Importantly, samples taken from peripheral villi at this time have revealed elevated levels of HSP70, which is in the heat-shock chaperone protein family and is upregulated during oxidative stress. It is also involved in the folding and refolding of aggregated or misfolded proteins [101], as well as nitrotyrosine residues indicative of peroxynitrite formation [98]. ROS and programmed apoptosis have also been found to play a role in maintaining homeostasis of the uterine endometrium during embryo implantation [102,103], and elevated levels of superoxide are believed to play a role in increasing vascular permeability during implantation [104]. Lastly, early placental maintenance is also dependent upon corpus luteum steroidogenesis, and both enzymatic and non-enzymatic antioxidants have been implicated in rescuing the corpus luteum from ROS-induced luteolysis [105,106,107,108,109].

Unfortunately, there is a paucity of data regarding how terrestrial, exogenous sources of oxidative stress affect this pro-oxidative state of early placentation, and there is even less regarding how cosmic radiation or microgravity may affect placentation [110]. Nevertheless, efforts are currently underway by these authors investigating such effects [111].

### 7.2. Oxidative Stress and Miscarriage

Among the numerous causes for early miscarriage, impaired placentation [112] and disruption of the physiological, oxidative damage-mediated process described above is frequently cited. Jauniaux et al. have observed similar elevations in HSP70 and nitrotyrosine in both the peripheral and central villi in placentas obtained after early miscarriage [98,113]. Furthermore, the apoptotic index is elevated and morphological evidence of syncytiotrophoblast sloughing is present, supporting a role for oxidative destruction of these tissues. Given the high metabolic rate of the placenta during early pregnancy, depletion of placental antioxidants has also been associated with early miscarriage [114]. Other studies have revealed decreased SOD expression and elevated lipid peroxidases in placental tissues following early miscarriage [106,107,115,116]. Further, polymorphisms in enzymatic antioxidants, as well as deficiency in selenium, have been associated with an increased risk of miscarriage [117,118,119,120,121,122]. Small studies have revealed that periconception selenium supplementation may decrease miscarriage rate in animal studies [123], and antioxidant supplementation with *N*-acetyl cysteine has improved the likelihood of successful human pregnancy following recurrent pregnancy loss [124].

### 7.3. Oxidative Stress and Embryopathy

Limited data exist directly linking oxidative stress with embryopathy, however, numerous insights can be made through the study of decreased antioxidant systems. One study has linked decreased maternal enzymatic antioxidant levels with birth defects [125]. Others have revealed that impaired SOD1 function alone [126], or maternal copper deficiency, which is a cofactor of SOD, have been linked with an increase in ROS and embryopathy [127,128]. Thus, more research is needed linking oxidative stress and embryopathy.

### 7.4. Oxidative Stress and Pre-Eclampsia

Pre-eclampsia is a common, and potentially severe, complication of pregnancy, characterized by elevated blood pressure, proteinuria, and evidence of end-organ damage (such as liver dysfunction, renal dysfunction, central nervous system dysfunction, thrombocytopenia, or pulmonary edema). Furthermore, worsening pre-eclampsia can lead to maternal seizures or stroke, and is associated with multiple adverse pregnancy outcomes, such as FGR, preterm birth, and placental abruption [129]. The pathophysiology is still not well-understood; however, there are clear links between this disorder, oxidative stress, and a systemic inflammatory response [92,125,130,131,132,133,134,135]. One explanation is that impairment of antioxidant activity during placentation may lead to an increase in lipid peroxidation and subsequent vascular endothelium damage [136,137]. Another similar explanation is that low-grade ischemia-reperfusion injury that has occurred secondary to abnormal remodeling of the spiral arteries during placentation leads to oxidative stress [2,138,139]. Lending some credence to this hypothesis is that the ischemia and reperfusion that occurs during labor also leads to a similar elevation in markers of oxidative stress and alterations in gene expression [140,141]. This placental oxidative stress then leads to the release of cytokines, angiogenic factors, and apoptotic debris into maternal circulation, which drive an inflammatory response [130]. Notably, the highest levels of oxidative stress markers are seen in pregnancies complicated by both pre-eclampsia and FGR [142,143,144], which are both believed to be caused by abnormal placental implantation and growth.

### 7.5. Oxidative Stress and Fetal Growth Restriction

While numerous etiologies for FGR exist, including chronic maternal disease, fetal chromosomal abnormalities, or fetal infections, placental insufficiency accounts for the majority of cases [129]. FGR is associated with numerous health risks for the infant later in life, such as metabolic syndrome, type II diabetes, and cardiovascular disease [145]. Multiple studies have found elevated markers of oxidative stress and evidence of lipid peroxidation in the maternal and fetal/neonatal blood [134,146,147,148,149], as well as decreased enzymatic antioxidant levels in neonates affected by FGR [150].

### 7.6. Oxidative Stress and Preterm Premature Rupture of Membranes

Oxidative stress has also been associated with PPROM, which is defined as a rupture of the amniotic sac before 37 weeks of gestation. PPROM carries risks of maternal and fetal infection, preterm delivery (PTD), and the risks of neonatal prematurity if a PTD were to occur [151]. A paucity of data exploring this link exists, however, in vitro models involving exposure of the chorio-amnion to superoxide have been shown to trigger upregulation of matrix metalloproteinase-9 and many inflammatory cytokines that predispose to PPROM [152]. Further, it is notable that numerous risk factors for PPROM, including cigarette smoking or drug use, vaginal bleeding, and the subsequent release of iron, as well as maternal or fetal infection, also increase ROS generation [153].

### 7.7. Oxidative Stress and Gestational Diabetes Mellitus

Gestational diabetes mellitus (GDM) is a condition in which pregnant patients with no prior history of diabetes will develop insulin resistance and glucose intolerance. Initial treatment involves changes in lifestyle, including eating habits and exercise, however, it often requires treatment with oral medications and/or insulin. Further, GDM carries numerous pregnancy risks to both the mother and fetus, and it is an important risk factor for the development of post-partum diabetes mellitus [154]. Both evidence of increased lipid peroxidation and increased activity/expression of antioxidants have been found in patients affected by GDM, raising concern for oxidative stress as a possible risk factor for, or etiology of, GDM [155,156,157].

## 8. Oxidative Stress and Infertility

Long-term oxidative stress has been associated with reduced follicle quality and decreased ovarian reproductive function in mice [158]. Further, oxidative damage has been linked with apoptotic cell death in ovarian follicles and granulosa cells that ultimately leads to ovarian aging [159]. Low-quality oocytes may be attributed to ROS [160,161], and heavy-ion radiation has even been associated with premature ovarian insufficiency (POI) [162]. However, POI is only one cause of infertility. Unfortunately, approximately 17% of couples suffer from infertility due to a number of reasons, including: ovulatory disorders, endometriosis-related infertility, tubal-factor infertility, male-factor infertility, and unexplained infertility [163]. While not deemed to be a direct causal agent, numerous studies have linked the disruption in physiological levels of ROS with reproductive dysfunction and infertility [164]. In fact, because ROS are so instrumental to physiological reproductive function, attempts have been made to determine the appropriate threshold values of ROS, above which may be pathological to fertility [165]. On the other hand, decreased enzymatic antioxidant levels [89,166,167], as well as decreased antioxidant micronutrients [168,169,170], have also been noted in patients affected by infertility. Lastly, though this review focuses on females, oxidative stress has also been linked with male-factor infertility [171].

## 9. Spaceflight and Ontogenesis

Traditionally, it has been assumed that an understanding of any organ, organism, or physiological system includes knowledge of its development. In the space life sciences, the value of ontogenetic knowledge has long been understood by researchers, but developmental investigations have been sporadic at best. Some relatively isolated studies into the effects of the space environment on the early development in non-mammalian systems, such as sea urchins, amphibians, fish, and birds, have not uncovered lethal or adverse effects on animal reproduction [172,173,174,175,176,177,178,179,180]. Mammalian reproduction and development during and following spaceflight have also been investigated, but here too, programmatic studies have not been supported, though they are sorely needed.

One small experiment did attempt mating two males with five females in space, however, no pregnancies resulted, and questions remain whether the failure may have been attributable to the cosmic environment or even an explanation as simple as imperfect housing [180,181,182,183]. In another study, mouse embryos were launched at the two-cell stage and developed for 4 days during the STS-80 mission. Upon return to Earth, none of the embryos showed any sign of further development or division [184]. The authors concluded that while the cosmic environment may have led to this result, environmental factors, such as vibration during spaceflight, may have also had an effect. A number of experiments have also focused on spermatogenesis to evaluate the functional ability of sperm. Rodent testes flown for short durations did not reveal morphological changes or impaired spermatogenesis [185,186]. More recent studies evaluated mouse spermatozoa that had been freeze-dried and stored on the International Space Station for 9 months. After thawing the spermatozoa, investigators found slightly more DNA damage to the spermatozoa and male pronuclei, however, this had no effect on fertility and fecundity [187].

The main corpus of space life sciences research on mammalian ontogenesis are derived from a series of spaceflight experiments performed over 25 years ago in association with the unmanned Cosmos Biosatellite program and the NASA-NIH (National Insitutes of Health) missions flown on the Space Shuttle. These international, multi-investigator studies evaluated pregnant rats sent into orbital spaceflight during time periods between completion of placental implantation and before delivery. Together, the results indicated that in the absence of Earth-normal gravity, and in the full context of spaceflight conditions, mammalian pregnancy could continue. Contrary to the predictions of some, rat dams that gestated in space and returned to Earth shortly before their due date, resulted in successfully vaginally-delivered litters of viable offspring [181,188,189], albeit with more labor contractions to complete the process [182]. The offspring in these studies displayed essentially normal morphology. Sensory testing revealed basic functions in each modality, although there was evidence that vestibular development was altered in the absence of normal linear and angular forces [190]. However, recovery of function did occur in the presence of Earth-normal gravity. Lastly, the rat dams returned to Earth not only ready to give birth, but also to lactate and express effective maternal behavior.

As part of the NASA-NIH experiments, suckling pups were launched with their nursing mothers [183]. For the NIH.R3 mission, pups and dams were launched on postnatal day 5, 8, or 14. All litters launched at 14 days of age survived and showed normal growth. Ninety percent of the pups launched at Day 8 survived, but their body weights were 25% less than the controls. Of the pups launched at Day 5, only 10% survived. During the NeuroLab mission, pups were launched at similar timepoints. Rat pups launched at 15 days of age survived the mission in good health. However, more than half of the pups launched at 8 days of age either showed failure to thrive or died [191]. The authors attributed the cause to malnutrition, dehydration, and hypothermia. Some remarkable feats of mother-offspring adaptive behaviors were video-recorded, but the vital importance of providing habitats with specialized features to enable each family to aggregate and maintain close proximity to each other was most apparent.

Clearly, further systematic studies evaluating the effects of microgravity and cosmic radiation on reproductive and ontogenetic processes are imperative. Such research has great value for fundamental physiologic knowledge in general, however, as we approach long-duration spaceflights that will also expose humans to higher levels of radiation, there is an even greater imperative to understand reproductive and developmental processes in the context of spaceflight.

## 10. Hormesis

As with many biological systems in which achieving balance is necessary, minor disturbances in the balance of ROS are likely to lead to homeostatic adaptations, whereas major perturbations may lead to irreparable cell damage and apoptosis. Hormesis refers to beneficial effects of stressors that induce a response that results in stress resistance [10]. Historically, many radiation studies have targeted large one-time dose responses, rather than the response to continuous low-dose radiation. With that said, numerous studies have found that lower doses of radiation may induce radioadaptive responses [192,193]. The most relevant of such studies involved exposure of pregnant mice to a priming dose of 10–13 mSv per day of gamma irradiation for 10 days from embryonic day 7–16. The pups were then born on embryonic day 18–19, and subsequently exposed to an additional large challenge dose of 2400 mSv on day-of-life 16–18, and euthanized for evaluation. When evaluating micronuclei as a marker of double-stranded DNA breaks, they found a significantly lower frequency of breaks in pups who were exposed to in utero low-dose radiation prior to the challenge dose [194]. The authors hypothesize that one explanation for these findings may be increased efficiency of DNA repair and oxidative stress metabolism following the original priming stressor [195].

## 11. Sex Differences

Since 1961, when the Soviet Union first launched a human into space, more than 550 humans have now orbited the Earth (https://www.worldspaceflight.com/bios/stats.php). Eleven percent have been women (https://history.nasa.gov/women.html). There is much to be learned about sex differences in physiology and behavior in that it is relevant to the understanding and management of health and disease. Thus, it is significant that since 2013, the number of females selected for NASA astronaut classes have been similar to the number of males selected, signaling the opportunity—as well as the need—for a deeper understanding of physiological adaptation to spaceflight in women [196].

Underscoring the value in better understanding the mechanisms of sex differences, is evidence that many human diseases are sex-specific, with regard to prevalence, age of onset, and/or severity [197,198,199], and some of these differences may be attributable to the antioxidant role that estrogen may play. Some examples include hypertension, hyperlipidemia, diabetes, and cardiovascular disease (CVD), for all of which oxidative stress has been implicated mechanistically [200,201,202]. In comparison to men at the same ages, CVD risk is reduced in pre-menopausal women with intact ovaries [203], and it has long been known that estrogen may play a cardio-protective role [204,205]. This is not true in post-menopausal women [206], when the loss of ovarian hormone production results in post-menopausal circulating estradiol levels that are even lower than those of age-matched men. Whereas testosterone has no antioxidant properties and is linked to increased susceptibility to oxidative stress [207] and reduced immunocompetence [208], estrogen reduces ROS production, and is a potent antioxidant and regulator of antioxidant genes [209]. Beyond the effects of estrogen, both human and animal studies have documented lower levels of ROS production and reduced susceptibility to the adverse effects of oxidative stress in females [210]. Important differences have included altered anti-oxidant enzyme activity levels, NADPH-oxidase levels (especially p47 and Nox levels), and angiotensin II levels [210].

In addition to its putative role in disease states, there is compelling evidence implicating oxidative stress in aging and biological sex. The longevity “gender gap” refers to the observation that females live about 10% longer than males in many species, including humans [209]. The role of oxidative stress as a consequence of normal metabolism has emerged as one feasible molecular mechanism to explain the aging process. Mitochondria are a major source of free radicals in cells that are sex-specific, generating 50% less hydrogen peroxide and higher levels of mitochondrial-reduced glutathione in females compared to males [211]. Mitochondrial dysfunction and associated oxidative stress have been implicated in cellular senescence, apoptosis, and aging [212]. Shortening of telomeres (the protective region of repetitive nucleotide sequences at the ends each chromosome) due to oxidative stress may underlie sex-specific differences in lifespan. Oxidation from ROS can result in the loss of telomeric base pairs, especially guanine [213], and cause cumulative damage to biomolecules [209]. Further, while breaks in the internal genome DNA are repaired quickly, repairs to equivalent breaks in telomeres are slow and less reliable, resulting in significant telomere attrition and cellular senescence [212].

Clearly, there is much to be learned about the association between oxidative stress and sex differences, and this knowledge can contribute to better management of both men and women in space. Further, by studying the differences between males and females during spaceflight, knowledge gained may foster improvements in biomedical practices on Earth.

## 12. Developmental Programming and Epigenetic Transgenerational Inheritance

Human epidemiological studies, combined with cellular, biochemical, and animal research, spawned the compelling concept of “fetal programming”—the idea that suboptimal maternal environments during early life can set the stage for chronic disease in adulthood [214,215]. Fetal programming is now viewed more broadly as “developmental programming” [216] with an even broader view that degradations in the early environment (in utero through adolescence) can have enduring consequences for metabolic, cardiovascular, and neuroendocrine pathophysiology across one’s lifespan [217,218,219]. Stress, parental care, nutritional status, and relatedly, gut microbiota, are some of the early influences that have been shown to enhance susceptibility to numerous physiological and mental disorders in later life [218,220,221,222]. Environmental factors (toxins, stress, infection, drugs, and alcohol) are known to similarly “program” neurodevelopment and result in long-term alterations in the brain, physiology, and behavior [223]. Many of these effects are sex-specific, affecting males and females differently [222,224].

Remarkably, early exposure to adverse nutritional conditions and behavioral stressors do not merely shape health and disease within a generation, but these phenotypes are transmitted across generations. Prenatal offspring of mothers who were pregnant during the Dutch Hunger Winter famine had increased adiposity and poor health [225], and children whose grandmothers participated in Ramadan fasting during pregnancy were lighter and had lower placental weights [226]. Epidemiological evidence also suggests that paternal lineage is an important determinant in transgenerational transmission of a programming phenotype. Second-generation (F2) offspring whose fathers were exposed to the Dutch Hunger Winter famine had a higher BMI compared to unexposed offspring [227]. Offspring of mice undernourished in utero had perturbed metabolic profiles that was maintained for 50 generations [228]. Maternal protein restriction in rats was followed by accelerated catch-up growth, decreased ovarian reserve, increased intra-abdominal fat mass, and accelerated ovarian aging in the second generation [229]. Infant maternal separation, a rodent paradigm of early life stress [230], led to impaired emotional behavior, executive cognitive function deficits, and increased acetylation of the histone H4K12 protein (acH4K12) in the forebrain neocortex in adulthood, which are also propagated across generations in mice. Further, learning experiences of adult animals prior to mating may influence their future offspring [231], and Dias et al. found that the mode of transmission involves transgenerational inheritance via alterations in the germ line. Astoundingly, the effects of parental stress in offspring and in the parental germline could also be reversed using extinction-based behavioral strategies [232]. These are epigenetic modifications (e.g. DNA methylation and histone modification), which are long-term, stable changes in gene expression that do not involve a change in gene sequences, and provide an important mechanistic explanation of programming effects.

Importantly, oxidative stress has been linked with developmental programming. Some exogenous sources of oxidative stress that have been evaluated are maternal protein restriction [233], and even general caloric restriction [234]. Further, mouse offspring derived from obese pregnancies demonstrate hepatic oxidative stress and mitochondrial dysfunction [235]. Other studies have revealed that rat offspring from pregnancies affected by placental insufficiency have shown impaired oxidative phosphorylation and accumulation of ROS in hepatic [236] and skeletal muscle mitochondria [237], as well as increased mitochondrial DNA mutations [238]. Lastly, rat offspring derived from dams that consumed a “junk food” diet showed evidence of increased oxidative stress and mitochondrial dysfunction [239]. Thus, ROS generated by a variety of intrauterine conditions may play a key role in epigenetic developmental programming of future generations.

The dramatic phenomena and associated mechanisms constituting this field further support the idea that prenatal deficits that appear mitigated or even rectified during early postnatal life, can nonetheless reappear later in life as an altered function. “Programming” has been a powerful metaphor. Nevertheless, it is important to recognize that unlike a true “program” in which specific information is encoded and “read out” faithfully, the actual mechanisms associated with prenatal influences exist as general, uninformed, probabilistic alterations in low-level molecular events. These important perturbations are expressed through webs of complexity, rather than as a reading of instructions with isomorphic expressions. The challenge has been, and remains, to identify and understand the intervening mechanisms. Oxidative stress is a strong candidate as an intervening mechanism, and it deserves scrutiny in a variety of settings, including spaceflight, for the many reasons articulated throughout the present review.

## 13. Frameworks for Viewing and Using Oxidative Stress for Better Understanding of Altered Reproduction and Development in the Space Environment

The present review reveals a largely unexplored landscape of basic biological processes that were adaptively shaped for life on Earth, but are now being increasingly challenged to function in the markedly “unearthly” environment of deep space. As an intellectual exercise, understanding how Earth-evolved adaptations perform in spaceflight environments is a stimulating pursuit—a veritable dream to a basic scientist. But there is imminent practicality to the endeavor. Long-duration space exploration by humans will soon begin, and we are facing challenges to human health, performance, safety, and ethical concerns for which we are ill-prepared. Chief among these challenges is understanding how to recognize, evaluate, manage, repair, and prevent maladaptive alterations in mammalian reproductive and developmental processes related to spaceflight.

Here, we have summarized a swath of observations and experimental findings that document the myriad of ways in which human bodies, brains, and behavior can change with exposure to microgravity and cosmic radiation—not to mention the combined contextual effects of confinement, isolation, restricted diet, and continual risk. Linking many of the relevant pathophysiological phenomena reviewed earlier in the present paper is oxidative stress. What might this mean for our questions about life in space? Here we briefly consider some possibilities:

### 13.1. Does Oxidative Stress That Is Associated with Spaceflight Lead to Alterations in Reproduction and Development?

Even at this early stage of knowledge-building, we think that there is good reason to consider oxidative stress as an underlying cause of some alterations in mammalian reproduction, as well as in perturbations of perinatal development. More specifically, phenomena that have been noted in spaceflight studies, or that may emerge as spaceflights, become longer and increasingly exposed to cosmic radiation, and may have causal connections to oxidative stress. The nature of these causal connections could take different forms. Damaging oxidative stress, rendered by unneutralized free-radicals, is, fundamentally, a by-product of normal cellular processes. Further, specific effects of cosmic radiation, general effects of microgravity (including its novelty for Earth-born organisms and its disruptions of Earth-adapted physiological homeostasis), and combinations of these factors can induce abundant ROS, cause inflammation, and trigger other pathways leading to tissue damage and dysfunction. In these ways, we must be on alert for oxidative stress associated with spaceflight-causing alterations in reproduction and development.

Mammalian reproduction and development involve processes of rapid and dramatic transformation, and thus present sequential “stages” of life. It is during such stages of dynamic change that systems are often most vulnerable to perturbation. As an example, the delicate balance between pro-oxidants and antioxidants that supports normal ovulation, early pregnancy, and early placental development, render these tissues at risk of dysfunction in the setting of spaceflight. It would be reasonable to hypothesize that oxidative stress may play a role in such dysfunction. Indeed, Tahimic and colleagues have discussed hypotheses regarding how both irradiation and HU hindlimb unloading have led to decreased levels of eNOS, which may then decrease the availability of nitric oxide (NO) and impair nitric oxide signaling. They then consider how this may lead to impaired endothelium-dependent vasodilation, as seen in a skeletal muscle arterial model [1]. As one example, to extend their hypotheses, if these spaceflight analogs could also lead to impaired vasodilation in the uterus, how might spiral arterial remodeling during early placentation be affected by the same stressors?

Here we hypothesize that oxidative stress, arising from spaceflight-like background forces, may exhibit the possibility of inducing altered developmental and reproductive processes at both cellular and systems levels. In these regards, space conditions and oxidative stress may have “causal” roles. It might be more precise to interpret oxidative stress as an intermediate step in a causal chain, but we can predict causal links.

### 13.2. Does Spaceflight-Related Alterations of Reproduction and Development Lead to Oxidative Stress?

At this early stage of knowledge-building, we do not have examples of causal pathways that begin with altered hormonal or tissue functions that lead demonstrably to excessive ROS which produce damage or disease associated with oxidative stress. Yet, given longer durations for spaceflight to induce and channel such alterations, it seems that such casual sequences could arise. We believe that the likelihoods are sufficient that health-related screening programs should be designed to monitor and detect such effects.

## 14. Oxidative Stress as a Biomarker for Altered Reproduction and Development Associated with Spaceflight

At this early stage of knowledge-building, the most appropriate and powerful role for oxidative stress may be as biomarkers, serving as signals to be vigilant for spaceflight-related alterations during reproduction and development. Much remains to be learned about specific mechanisms underlying any of the myriad effects of oxidative stress on numerous levels (cellular, to systems, to organismal levels) in relation to spaceflight. We are similarly challenged to explain causes of the phenomena. Nevertheless, a role as a biomarker need not denote a role as either cause or consequence. To be useful, it need only be a correlate or a predictor. Of course, understanding the mechanisms by which such correlations arise is an important addition that can help understand interpretative parameters, but as long as the correlation is good between oxidative stress and the appearance of a phenotypic change, there is justification of recognizing their utility as biomarkers.

The present review comprises numerous examples and findings that underscore the potential for utilizing biomarkers of oxidative stress in the evaluation of altered processes of reproduction or development. Rather than recite the many candidate examples, the interested reader is encouraged to consult each of the sections of interest and identify relevant potential biomarkers. Appropriate next steps would include examinations of the specificity of such effects, for it might be that the appearance of excessive ROS is too general to be a good predictor or marker for a specific outcome, unless it is accompanied by an additional signal or condition. Similarly, we would benefit from identifying target tissues that would provide accurate assays for reproductive or developmental perturbation. Nevertheless, it appears to us that there is ample evidence to pursue the next, early phases of oxidative stress biomarker evaluation in the context of spaceflight. The present review helps set the stage for such considerations.

## 15. Treatment Considerations

For spaceflight, numerous antioxidants have been evaluated or considered. An antioxidant diet cocktail (AOX) composed of *N*-acetyl cysteine, ascorbic acid, l-selenomethionine, dihydrolipoic acid, and vitamin E, has been shown to protect a variety of tissues from ionizing radiation [240,241,242]. Use of α-lipoic acid has been shown to decrease inflammation and acute, radiation-induced bone loss [80,243], while dried plum has also been shown to decrease bone loss in ovariectomized mice [83,244,245,246]. Nonsteroidal anti-inflammatory drugs [247,248], aspirin [249], and dihydrolipoic acid (DHLA) have also been shown to have antioxidant properties [250,251]. However, while many studies have evaluated a single organ system’s response to antioxidants, an ideal regimen would provide protection of multiple organ systems. Furthermore, given the close interaction between oxidative stress and ER stress, therapies targeting both of these processes simultaneously should be considered [14].

Regarding mitigation of oxidative stress on reproductive outcomes, antioxidant vitamins and supplements have been associated with decreased miscarriage risk and improved birth outcomes [170,252,253]. However, high vitamin E supplementation has also been associated with FGR [254]. Further, with the exception of aspirin, antioxidants have not been beneficial for preventing pre-eclampsia once pregnancies have been established [255,256,257], but have decreased the risk of pre-eclampsia when taken pre-pregnancy [258,259]. In light of these findings, more research is required to determine the timing and dosing of supplemental antioxidants during the reproductive period, and/or their potential teratogenicity.

## 16. Concluding Statements & Future Endpoints to Be Explored

ROS can be used for both physiological and pathological ends, and achieving a pro-oxidant/antioxidant balance in reproductive tissues, especially during pregnancy, is critical for normal early development, as well as mitigating morbidity during pregnancy.

Placental oxidative stress is clearly associated with multiple adverse pregnancy outcomes, and impaired trophoblast invasion and conversion of the maternal spiral arteries appears to be mechanistically linked. If microgravity and cosmic radiation were to shift the balance further towards a pro-oxidative state during pregnancy, we hypothesize that they may increase the risks of miscarriage, PPROM, preterm birth, FGR, pre-eclampsia, and gestational diabetes. Further, these insults may impair fertility to some extent as well. Unfortunately, there is a paucity of data evaluating reproductive endpoints following these exposures. Thus, we recommend animal studies aimed at evaluating terrestrial analogs of continuous low-dose exposure to protons, heavy ions, or neutrons and/or hindlimb unloading, with an interest in evaluating markers of placental oxidative stress and expression of ROS-scavenging and ROS-producing genes. Follow-up studies can again evaluate such endpoints in the setting of antioxidant-based countermeasures, as discussed above. Furthermore, continued evaluation of the effects of these exposures on normal menstrual cycling and ovulation are needed. Lastly, oxidative stress rarely occurs in isolation, and pathology arising in the setting of ROS may also be occurring alongside ER stress and other forms of cell stress. Thus, studies designed to evaluate such markers in concert may continue to grow our understanding of how cosmic exposures may affect reproductive outcomes.
